# Effects of Homogeneous Doping on Electron–Phonon Coupling in SrTiO_3_

**DOI:** 10.3390/nano15020137

**Published:** 2025-01-17

**Authors:** Minwoo Park, Suk Bum Chung

**Affiliations:** 1 Department of Physics and Natural Science Research Institute, University of Seoul, Seoul 02504, Republic of Korea; minwoo21@uos.ac.kr; 2 School of Physics, Korea Institute for Advanced Study, Seoul 02455, Republic of Korea

**Keywords:** electron–phonon coupling, electron pairing interaction, polar phonon modes, doped quantum paraelectric, dilute superconductor

## Abstract

Bulk n-type SrTiO_3_ (STO) has long been known to possess a superconducting ground state at an exceptionally dilute carrier density. This has raised questions about the applicability of the BCS-Eliashberg paradigm with its underlying adiabatic assumption. However, recent experimental reports have set the pairing gap to the critical temperature ($T_{c}$) ratio at the BCS value for superconductivity in Nb-doped STO, even though the adiabaticity condition the BCS pairing requires is satisfied over the entire superconducting dome only by the lowest branch of optical phonons. In spite of the strong implications these reports have on specifying the pairing glue, they have not proved sufficient in explaining the magnitude of the optimal doping. This motivated us to apply density functional theory to Nb-doped STO to analyze how the phonon band structures and the electron–phonon coupling evolve with doping. To describe the very low doping concentration, we tuned the homogeneous background charge, from which we obtained a first-principles result on the doping-dependent phonon frequency that is in good agreement with experimental data for Nb-doped STO. Using the EPW code, we obtain the doping-dependent phonon dispersion and the electron–phonon coupling strength. Within the framework of our calculation, we found that the electron–phonon coupling forms a dome in a doping range lower than the experimentally observed superconducting dome of the Nb-doped STO. Additionally, we examined the doping dependence of both the orbital angular momentum quenching in the electron–phonon coupling and the phonon displacement correlation length and found the former to have a strong correlation with our electron–phonon coupling in the overdoped region.

## 1. Introduction

One overarching theme of STO physics is the effect of its proximate continuous ferroelectric (FE) transition [1,2]. The effect is quite standard and well understood regarding some standard aspects, such as the high dielectric constant and the polar soft phonon mode [3,4,5,6]. More recently, there has been much debate on the possible relation between the proximate FE transition and the superconducting mechanism [7,8,9,10,11,12,13,14,15,16,17,18] of the dilute n-type bulk [19,20]. This superconductivity has long retained its notoriety for both being the first discovered instance to exhibit a doping-dependent superconducting dome and occurring at a carrier density lower than any other bulk superconductor, except the recently discovered doped Bi [21]. Given such prominent unconventional features, the results of optical conductivity measurements [22] and the tunneling spectroscopy measurements [10,13] for the Nb-doped STO superconducting phase in the last few years have been quite surprising, revealing the pairing gap-to-$T_{c}$ ratio to be at the BCS value for most of the superconducting dome. They raise the possibility of STO superconductivity, with all its unconventional features, arising from a BCS-type pairing mechanism, which is understood to impose the adiabatic criterion, i.e., the pairing interaction whose frequency is much smaller than the electron Fermi energy. A pairing interaction that can intrinsically satisfy the adiabatic criterion is provided by the proximity to a continuous FE transition, where the lowest polar phonon mode, called the TO1 mode, softens. In the case of STO, it is known that this is the only optical phonon mode that satisfies the adiabatic criterion throughout the superconducting dome [13].

While the phonon-mediated BCS pairing scenario offers some key qualitative explanations for the STO superconducting dome, it is not intrinsically sufficient in explaining the observed value of the optimal doping. It is true that within this scenario, the pairing suppression in the overdoped region can be explained [13,15,23] by the experimentally observed hardening of TO1 phonons with increasing doping concentration [4,24], but understanding how the optimal doping occurs at relatively dilute doping and, thus, limits the optimal critical temperature remains a challenge. Hence, the simplest models with electronic coupling to TO1 phonons [15,25] sought to address the emergence of the $T_{c}$ dome structure rather than to make good order-of-magnitude estimates for the optimal doping.

Within the framework of the phonon-mediated BCS pairing scenario, different theories have been proposed for the mechanism that limits the optimal doping. According to one recent theoretical proposal [25], this suppression can be attributed to attenuation of the effective electron–phonon matrix element from the orbital angular momentum (OAM) quenching of the Ti $t_{2g}$ orbitals at a higher doping concentration. In this proposal, the doping evolution of the phonon dispersion is relevant only for increasing the minimum frequency. In contrast, a recent inelastic neutron scattering experiment on n-type STO led to the suggestion [26] that the superconducting critical temperature $T_{c}$ is closely correlated with $k_{F}\ell_{0}$, where $\ell_{0}$ is the TO1 phonon displacement correlation length, and ${\left(k_{F}\ell_{0}\right)}^{3}$, therefore, would be proportional to the number of electrons within the range of the TO1 phonon-mediated interaction. These two proposed mechanisms are not equivalent, despite both arising from the phonon-mediated pairing scenario. The former proposal is strongly dependent on the orbital symmetry of the Ti $t_{2g}$ orbital, but the phonon dispersion does not feature beyond the frequency minimum; for the latter proposal, the exact converse is the case. Finally, it should be pointed out that the latter proposal does not have any particular dependence on the form of the electron–phonon coupling.

Given the need for a better understanding of the mechanism for limiting the optimal doping within the framework of phonon-mediated superconductivity, first-principle calculation of both the phonon band structure and the electron–phonon coupling in STO is a natural and necessary step in developing the full theory of its superconductivity. Indeed, first-principles calculations of the doping-dependent phonon band structure and electron–phonon coupling have been carried out in KTaO_3_ [27], another cubic perovskite transition metal oxide that is in the vicinity of the continuous ferroelectric transition. We report in this paper both phonon-mode-resolved linear electron–phonon coupling $\lambda_{q,\nu }$ and its summation over the first Brillouin zone (BZ) λ, obtained using the first-principles calculation based on density functional theory for cubic SrTiO_3_ over a range that covers the entire experimentally reported superconducting dome. The main findings of our calculations are that (i) we find a well-defined dome for λ with an optimal doping about one order of magnitude smaller than that of the superconducting dome; (ii) the λ suppression in the overdoped region can be closely tracked with OAM quenching, which can be expected from the linear coupling of electrons to the polar phonon mode [12,15,23,25]; (iii) $k_{F}\ell_{0}$ suppression is not evident on the overdoped side where λ is strongly suppressed.

## 2. Method

Using density functional theory (DFT), we performed calculations as implemented in the Quantum ESPRESSO package v.7.1 [28,29,30]. We employed full relativistic local density approximation (LDA) using Perdew–Zunger (PZ) parameterization for the exchange-correlation energy functional with the projector augmented wave method (PAW) in pslibrary [31,32,33]. The kinetic energy cutoff for wavefunctions was 60 Ry (816 eV). The 1st BZ integration was performed using the Monkhorst–Pack scheme with 16 × 16 × 16 k-point sampling [34]. Geometric optimization was carried out until the Hellmann–Feynman force acting on each atom was smaller than 0.1 meV/Å, from which we found the STO lattice constant to be well approximated by $a_{0}$ = 3.8565 Å for the three lowest doping values; for the higher doping values, where the results were much less sensitive to the lattice constant, we approximated the lattice constant as $a_{0}$ = 3.8600 Å (see Supplementary Section S3 for details). We achieved the doping effect with the background charge (called ‘jellium’) method using Gaussian smearing with a 0.1 meV width for five different doping levels in the range from 0.0001 to 0.02 electrons per unit cell (e/u.c.), which covers all but the upper critical doping of the experimentally measured superconducting dome [13,35]. The phonon dispersion was calculated on a 4 × 4 × 4 q-point mesh; 200 q-points were employed between high-symmetry points. We note that our phonon dispersion calculation considers doping far more dilute than some recent first-principles calculations, e.g., Ref. [36], where the VASP code was used.

The electron–phonon coupling effect was calculated with the EPW 5.5 code in the Quantum ESPRESSO package [37,38]. The relevant electronic bands in SrTiO_3_ are the three Ti-3d$t_{2g}$ bands, which are described by maximally localized Wannier functions using the Wannier90 code [39] within the EPW code, including Ti spin-orbit coupling, for which we obtain ξ = 20 meV, as shown in Supplementary Section S1. The electron–phonon matrix was calculated on coarse 16 × 16 × 16 k-points and 4 × 4 × 4 q-points and then interpolated onto a fine grid. We mixed two fine q-point meshes for efficient calculation to obtain the electron–phonon coupling constant λ near the Γ point. This special q-mesh is a combination of two grids, an 11 × 11 × 11 grid including a Γ point range from −0.5 to 0.5 in units of 2π/$a_{0}$, and a 20 × 20 × 20 Monkhorst–Pack grid range from −0.1 to 0.1 in units of 2π/$a_{0}$. For the mode-resolved electron–phonon coupling strengths, 200 q-points were again employed between each cubic high-symmetry point. The well-known splitting between the longitudinal (LO1) and transverse (TO1) branches of the lowest optical phonon is expected to evolve, and possibly attenuate, with doping due to its effect on charge screening [27]. To the best of our knowledge, a method to compute electron–phonon coupling while obtaining the Born effective charges and dielectric constants needed for LO–TO splitting in doped insulators has not been implemented. We leave this as a future challenge and ignore LO1/TO1 splitting altogether.

## 3. Results

### 3.1. Phonon-Mode-Resolved Electron–Phonon Coupling

We first give a summary of the standard formalism [37,40] we used to calculate the mode-resolved linear electron–phonon coupling strength, the key ingredient of which is the imaginary part of the phonon self-energy of the branch ν at momentum q to the one-loop order due to electron–phonon coupling:

(1) Πq,ν″=2Im∑m,n,k|gmn,νSE(k,q)|2fn,k−fm,k+qϵm,k+q−ϵn,k−ωq,ν−iδ=2π∑m,n,k|gmn,νSE(k,q)|2(fn,k−fm,k+q)×δ(ωq,ν+ϵn,k−ϵm,k+q),

where $\omega_{q,\nu }$ is the phonon frequency, and $\epsilon_{n,k}$ the electronic band energy for band *n* at momentum k. The electron–phonon matrix element here is given by

(2) $$g_{mn,\nu }^{\mathrm{SE}}\left(k,q\right)=\sqrt{\frac{\hbar }{2m_{0}\omega_{q,\nu }}}\langle m,k+q|\partial_{q,\nu }V|n,k\rangle .$$

where $m_{0}$ is the effective mass of the phonon mode, |n,k〉 the electronic Bloch state, and $\partial_{q,\nu }V$ the derivative of the self-consistent ionic potential *V* with respect to the collective ionic displacement of the phonon mode q,ν. The electron–phonon coupling strength for the phonon mode q,ν is proportional to Equation (1):

(3) $$\lambda_{q,\nu }=\frac{2}{\pi N_{F}}\frac{\Pi_{q,\nu }^{\prime \prime }}{\omega_{q,\nu }^{2}}$$

where $N_{F}$ is the electronic density of states at the Fermi level. The electron–phonon coupling strength is simply the summation of $\lambda_{q,\nu }$ over all phonon modes and the entire first BZ, i.e., $\lambda =\sum_{q,\nu }\lambda_{q,\nu }$.

**Figure 1:**
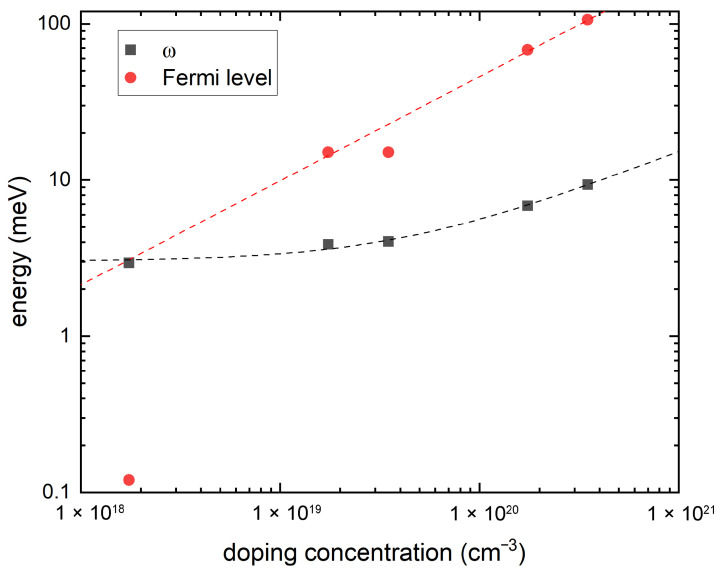
Doping dependence of the polar soft mode energy at Γ (black dots) and the Fermi energy (red dots). The black dotted curve shows the $\omega^{2}\left(n\right)=\omega_{0}^{2}+\gamma_{n}n$ fitting and the red dotted curve $E_{F}=\hbar^{2}{\left(3\pi^{2}n\right)}^{2/3}/2m^{*}$, with $m^{*}$ being approximately 1.65 times the free electron mass.

**Figure 2:**
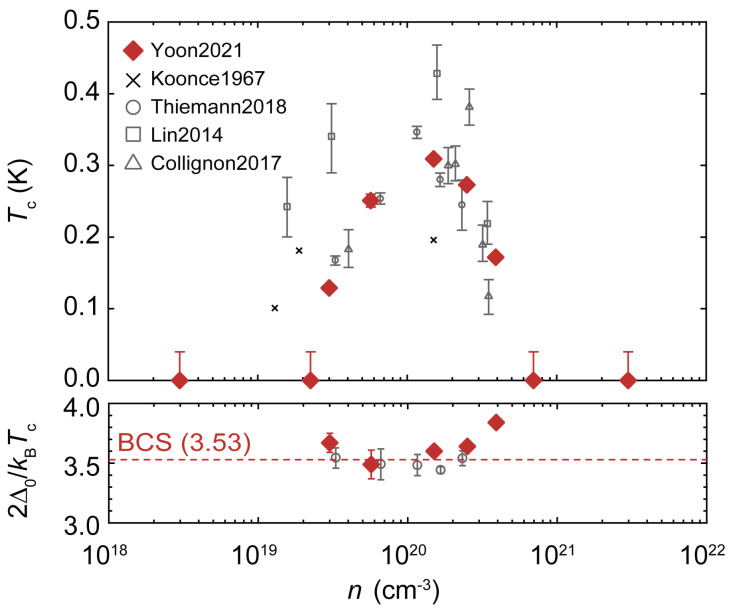
Superconducting critical temperatures and pairing gaps as a function of doping concentration for Nb-doped STO from Ref. [13]. The top plot shows the resistive transitions observed by Yoon et al. [13], Koonce et al. [20], Thiemann et al. [22], Lin et al. [41], and Collignon et al. [42], while the horizontal dashed red line of the bottom plot indicates the BCS value 2$\Delta_{0}$/k_B_T_c_ = 3.53.

Given that $\lambda_{q,\nu }$ can meaningfully quantify the electronic interaction mediated by phonons, i.e., without any vertex correction, only when the modes are adiabatic compared to the Fermi energy, we plot in Figure 1 the doping evolution of the frequency of the polar soft mode at Γ together with the Fermi energy. Both are obtained from our first-principles calculations, with the latter determined to be the energy at which the electron occupation number falls to 1% of the occupation number at Γ. We see that our polar soft mode at Γ hardens to 9.35 meV for 0.02 e/u.c. from 2.95 meV for 0.0001 e/u.c., and this can be fitted to $\omega_{0,\nu }^{2}\left(n\right)=\omega_{0}^{2}+\gamma_{n}n$, where $\gamma_{n}$= 2.2$\;\times \;10^{-19}$ meV^2^· cm^3^ and $\omega_{0}^{2}$ = 9.1 meV^2^; for comparison, applying the same fitting to experimental phonon data is known to give $\gamma_{n}$ = 1.8 ×$10^{-19}$ meV^2^· cm^3^ and $\omega_{0}^{2}$ = 1 meV^2^ at 4K [4,35]. Meanwhile, we find that our results for the Fermi energy fit very well to the free electron formula except for the lowest doping at 0.0001 e/u.c., or equivalently, $n=1.7\times 10^{18}$ cm^−3^; the discrepancy here may be attributed to the finite mesh size causing worse problems when $E_{F}$ becomes very small. In general, we find that the polar soft mode can be taken to be adiabatic for the doping concentration $n>10^{19}$ cm^−3^. The experimentally measured superconducting dome, as shown in Figure 2, has a lower critical doping larger than $10^{19}$ cm^−3^, which means that the relevant underdoped region is well within the adiabatic regime. However, we also carried out calculations for the doping of $n=1.7\times 10^{18}$ cm^−3^; as a doping value outside the adiabatic regime, it can be used as a negative benchmark, i.e., to examine what happens to our first-principles calculation when the adiabaticity condition breaks down.

**Figure 3:**
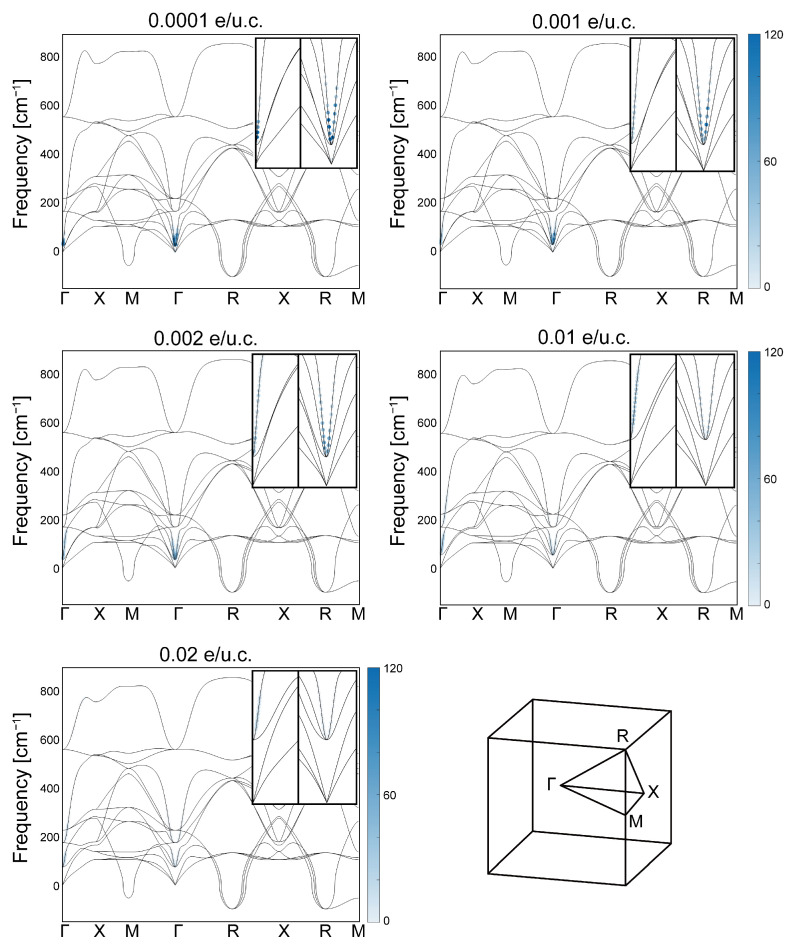
Phonon-mode-resolved electron–phonon coupling $\lambda_{q,\nu }$ along the high-symmetry lines of the first BZ at doping concentrations ranging from 0.0001 e/u.c. to 0.02e/u.c., i.e., approximately 1.7 ×$10^{18}$ cm^−3^ to 3.4 ×$10^{20}$ cm^−3^, plotted on top of phonon frequencies (drawn as narrow black curves); note that the frequency 1 cm^−1^ corresponds to approximately 0.124 meV. All plots are on the same scale and share the same colorbar for the dimensionless $\lambda_{q,\nu }$. The insets in the top right corners of each subplot show a zoomed-in part of the area around Γ towards the X, M and R points.

We show in Figure 3 the mode-resolved electron–phonon coupling $\lambda_{q,\nu }$, together with the full phonon spectra, along the high-symmetry direction of the first BZ for five different doping values. We first note that our geometrical optimization removed the instability toward uniform polar distortion, leaving no imaginary frequency near Γ at any doping values; *cf.* Ref. [36]. For all doping levels, we emphasize that the $\lambda_{q,\nu }$ distribution is massively concentrated in the polar soft mode near Γ. Although there is a secondary contribution from the higher-energy optical mode around Γ, we did not find any significant contribution from either the acoustic mode across the entire first BZ or any modes around any other high-symmetry points in the first BZ. We therefore argue that instabilities around high-symmetry points other than Γ can be ignored for our purposes.

### 3.2. Analysis of Electron–Phonon Coupling

**Figure 4:**
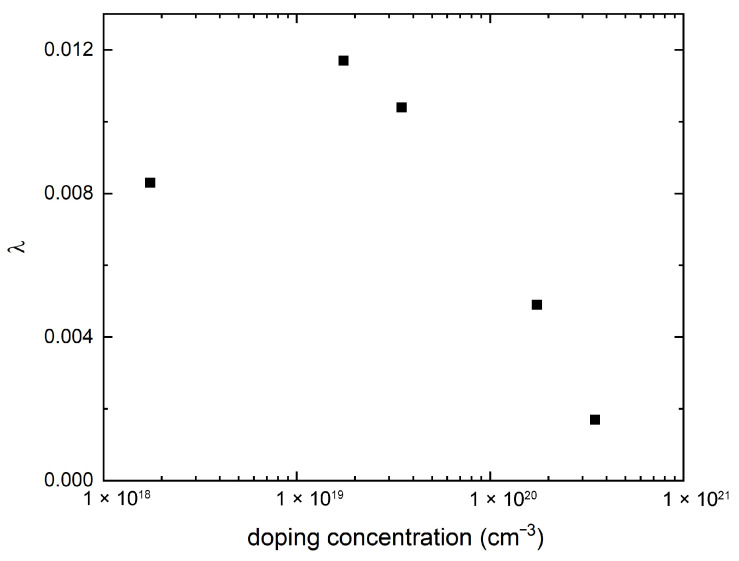
The doping evolution of the total electron–phonon coupling strength $\lambda =\sum_{q,\nu }\lambda_{q,\nu }$, with the summation carried out over all phonon modes and the entire 1st BZ.

The electron–phonon coupling strength λ, obtained by integrating $\lambda_{q,\nu }$ over the entire first BZ, is shown as a function of the doping concentration in Figure 4. A well-defined dome structure is discernible in the calculated λ, with the optimal doping occurring around $n=1.7\times 10^{19}$ cm^3^; for comparison, the optimal doping for $T_{c}$ in the Nb-doped STO [13,22] occurred around $n=1.5\times 10^{20}$ cm^−3^, as shown in Figure 2. Closely related to the optimal doping is the value of the upper and lower critical dopings. The data in Figure 4 can be reasonably extrapolated to obtain $n\approx 5\times 10^{20}$ cm^−3^ for the λ upper critical doping, which can be compared to the $T_{c}$ upper critical doping of approximately $n=7\times 10^{20}$ cm^−3^ [13] shown in Figure 2. Evidently, there is better agreement for the upper critical doping compared to the optimal doping, even if the discrepancy in the latter is still within an order of magnitude. In contrast, the λ lower critical doping falls outside the doping range of Figure 4, which is a more marked discrepancy compared with the $T_{c}$ lower critical doping of $n=2.5\times 10^{19}$ cm^−3^. Indeed, we note here that due to the λ optimal doping being considerably smaller than the $T_{c}$ optimal doping, the λ dome does not fall entirely within the adiabatic regime, as can be seen from Figure 1.

**Figure 5:**
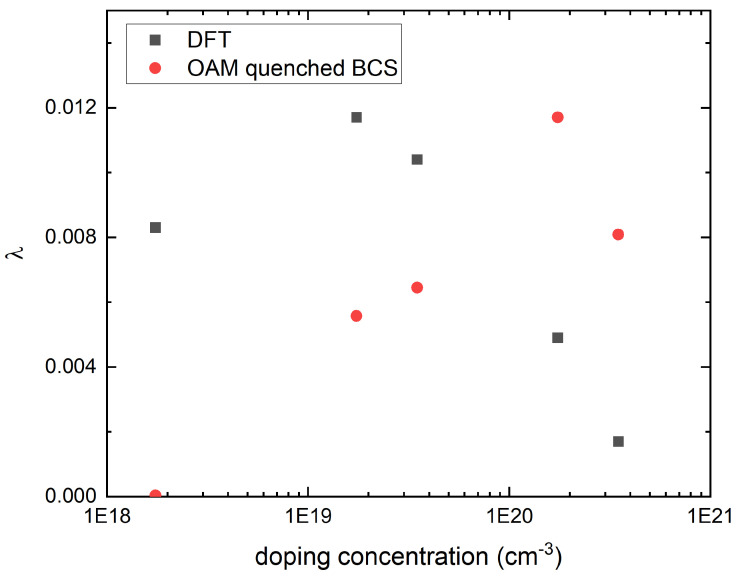
The BCS λ that includes the orbital angular momentum quenching effect, marked in red, is compared to the λ obtained from our first-principles calculation.

Given that our first-principles calculations fully incorporate the STO crystalline structure, Figure 5 plots a relevant comparison between our λ and the BCS eigenvalue that incorporates the crystalline (and hence, orbital) symmetry effect. One such example is the recently discussed model [25] for the OAM quenching of the electron–phonon coupling at a higher doping concentration. It has been pointed out previously [15,23] that electronic coupling to the polar phonons has the strongest effect in the region close to the Ti $t_{2g}$ orbital energy crossing curves. But when the inter-orbital next-nearest neighbor hopping allowed by the orbital symmetry is accounted for, the resulting orbital hybridization quenches the OAM. This quenching effect, as discussed in Ref. [25], modifies the BCS pairing eigenvalues of Refs. [13,15] into

(4) $$\lambda_{\mathrm{OAM}}\propto \frac{n^{1/3}}{\omega^{2}}\frac{\sin^{2}\left(k_{F110}a\right)}{1+{\left[\frac{4t_{4}\sin^{2}\left(k_{F110}a\right)}{\xi }\right]}^{2}},$$

where the Fermi wave vector $k_{F110}$ in Figure 5 is determined by the same method as the $E_{F}$s of Figure 1; ω is the polar soft mode frequency at Γ, as plotted in Figure 1; $t_{4}$ = 40 meV is the inter-orbital next-nearest neighbor hopping between Ti $t_{2g}$ orbitals; and ξ = 20 meV is the Ti atomic spin-orbit coupling (details on the first-principles calculation of $t_{4}$ and ξ are given in Supplementary Section S1); for convenience, the proportionality constant of Equation (4) is set so that the $\lambda_{\mathrm{OAM}}$ maximum would match the maximum of the DFT λ. We see that both the optimal doping and the upper critical doping of the $\lambda_{\mathrm{OAM}}$ are larger, but within an order of magnitude, than those of the λ dome, making them closely match those of the $T_{c}$ dome shown in Figure 2. In the underdoped region, however, Figure 5 shows the difference between the $\lambda_{\mathrm{OAM}}$ dome and the λ dome to be more substantial, in particular due to the occurrence of the lower critical doping for the former around $n=1.7\times 10^{18}$ cm^−3^.

**Figure 6:**
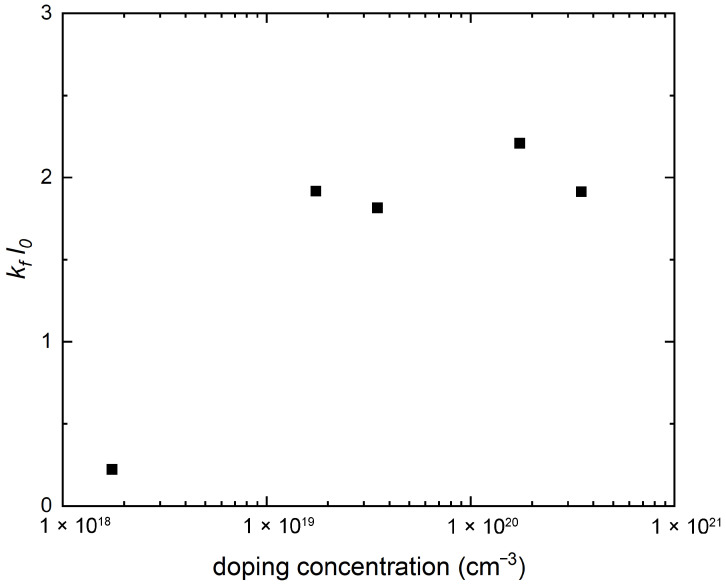
The doping evolution of $k_{F}\ell_{0}$, where $\ell_{0}\equiv v_{p}/\omega$ is the polar soft mode correlation length.

Lastly, in Figure 6, we examine through the first-principles calculation the doping evolution of the product of $k_{F}$ and the polar soft mode correlation length $\ell_{0}$, which, as pointed out recently [26], can tell us how many electrons are within the range of phonon-mediated interaction. Following Ref. [26], we concentrated on the interaction effect on the Fermi surface around the [110] direction where the linear electron–phonon coupling is expected to be strongest [12,15,25]. This $\ell_{0}\equiv v_{p}/\omega$ along the [110] direction is defined from the dispersion along the [110] direction $E^{2}\left(q\right)=v_{p}^{2}q^{2}+\omega^{2}=\omega^{2}\left(1+q^{2}\ell_{0}^{2}\right)$ of the mode with the strongest electronic coupling. As shown in Figure S2 in Supplementary Section S2, our first-principles calculation shows $\ell_{0}$ decreasing with doping, mainly due to ω increasing with doping, as shown in Figure 1, but also due to $v_{p}$ slightly decreasing with doping. As plotted in Figure 6, the doping evolution of $k_{F}\ell_{0}$, where $k_{F}$ is the $k_{F110}$ of Equation (4), also shows a dome-like structure. Although the lower critical doping is clearly discernible for this dome, the optimal doping or the upper critical doping cannot be determined, as $k_{F}\ell_{0}$ essentially does not decrease in the overdoped region. Within the context of our calculation, the $\lambda_{\mathrm{OAM}}$ and the $k_{F}\ell_{0}$ domes of Figure 5 and Figure 6 clearly do not coincide, the former (latter) being shifted up (down) in the doping range when compared to the experimental $T_{c}$ dome of Figure 2.

## 4. Discussion and Conclusions

We have shown in Figure 3 and Figure 4 our first-principles calculation of doping-dependent electron–phonon coupling. At all doping concentrations, the distribution of the mode-resolved electron–phonon coupling, $\lambda_{q,\nu }$ as shown in Figure 3, is strongly concentrated in the polar soft mode around Γ, a possible indication that these modes mediate the pairing interaction. A comparison between the dome for the integrated electron–phonon coupling λ shown in Figure 4 and the experimental $T_{c}$ dome shown in Figure 2 shows a shift to a lower doping range of the former, with a significant enhancement in the underdoped region.

One possible way to expand upon our results is to find a way to incorporate into our first-principles calculations the doping evolution of the energy splitting of the polar modes at Γ between the longitudinal mode (LO1) and the transverse mode (TO1). According to infrared [5] and hyper-Raman [6] experiments on undoped STO bulk, the LO1 mode frequency at Γ is greater than that of the TO1 mode by about 20.3 meV, or equivalently, 163 cm^−1^, according to the unit used in Figure 3. Given that at similar energy, our Figure 3 shows $\lambda_{q,\nu }$ to be insignificant, it may be reasonable to postulate that this splitting would suppress the electronic coupling to the LO1 phonons in the underdoped region. However, our DFT calculations ignored LO1/TO1 energy splitting, and the resulting $\lambda_{q,\nu }$ was found to be approximately two orders of magnitude larger for the LO1 mode. This suggests that the large λ in the underdoped region in Figure 4 would have been sharply attenuated upon the inclusion of LO1/TO1 splitting and that our calculation is more physically relevant in the overdoped region. It should be noted that our experiments found the LO1/TO1 splitting to remain substantial (∼10 meV) in the overdoped region, yet the upper critical doping of our Figure 4 approximates the upper critical doping of $T_{c}$, as shown in Figure 2; this may indicate the effect OAM quenching has on any polar displacements.

Lastly, it is worth noting that $k_{F}\ell_{0}$ is intrinsically independent of any orbital symmetry or any particular form of electron–phonon couplings, and consequently, is potentially useful as diagnostic for non-linear electron–phonon coupling. Indeed, the two-phonon mediated pairing interaction [16,43,44] would plausibly be more relevant for a larger $1/k_{F}\ell_{0}$, i.e., a larger number of phonons within the average distance between electrons; this would occur in the underdoped region according to our calculations for Figure 6. Devising a DFT calculation for doping-dependent two-phonon coupling analogous to our linear electron–phonon coupling would be an interesting future challenge, and it would be worth comparing such calculations with the DFT $k_{F}\ell_{0}$ result.

## Data Availability

Relevant data in this paper are available upon reasonable request.

## Supplementary Materials

The following supporting information can be downloaded at: https://www.mdpi.com/article/10.3390/nano15020137/s1, Figure S1: Calculated band structure and tight-binding fitting near Γ point with 0.002e/u.c.; Figure S2: The doping evolution of the velocity $v_{p}$ (upper) and the correlation length $\ell_{0}\equiv v_{p}/\omega$ (lower), respectively, of the polar soft mode. The former is obtained from fitting to $E^{2}\left(q\right)=\omega^{2}+v_{p}^{2}q^{2}$ along the [110] direction, and the latter from ω taken from Figure 2 of the main text; Figure S3: The doping evolution of the lattice parameter. As explained in Sec. II of the main text, we show the lattice constant for which the Hellmann–Feynman force, i.e., stress, of the cubic lattice is minimized. For comparison, the values of $a_{0}=3.8565Å$ and $a_{0}=3.8600Å$ are indicated by the two sets of horizontal dashed segments.

## References

[1] Müller K.A., Burkard H. (1979). SrTiO_3_: An intrinsic quantum paraelectric below 4 K. Phys. Rev. B.

[2] Haeni J.H., Irvin P., Chang W., Uecker R., Reiche P., Li Y.L., Choudhury S., Tian W., Hawley M.E., Craigo B. (2004). Room-temperature ferroelectricity in strained SrTiO_3_. Nature.

[3] Yamada Y., Shirane G. (1969). Neutron scattering and nature of the soft optical phonon in SrTiO_3_. J. Phys. Soc. Jpn..

[4] Bäuerle D., Wagner D., Wöhlecke M., Dorner B., Kraxenberger H. (1980). Soft modes in semiconducting SrTiO_3_: II. The ferroelectric mode. Z. Phys. B Condens. Matter.

[5] Vogt H., Rossbroich G. (1981). Accurate determination of the far-infrared dispersion in SrTiO_3_ by hyper-Raman spectroscopy. Phys. Rev. B.

[6] Kamaras K., Barth K.L., Keilmann F., Henn R., Reedyk M., Thomsen C., Cardona M., Kircher J., Richards P., Stehlé J.L. (1995). The low-temperature infrared optical functions of SrTiO_3_ determined by reflectance spectroscopy and spectroscopic ellipsometry. J. Appl. Phys..

[7] Rowley S., Spalek L., Smith R., Dean M., Itoh M., Scott J., Lonzarich G., Saxena S. (2014). Ferroelectric quantum criticality. Nat. Phys..

[8] Edge J.M., Kedem Y., Aschauer U., Spaldin N.A., Balatsky A.V. (2015). Quantum Critical Origin of the Superconducting Dome in SrTiO_3_. Phys. Rev. Lett..

[9] Rischau C.W., Lin X., Grams C.P., Finck D., Harms S., Engelmayer J., Lorenz T., Gallais Y., Fauqué B., Hemberger J. (2017). A ferroelectric quantum phase transition inside the superconducting dome of Sr_1-x_Ca_x_TiO_3-δ_. Nat. Phys..

[10] Swartz A.G., Inoue H., Merz T.A., Hikita Y., Raghu S., Devereaux T.P., Johnston S., Hwang H.Y. (2018). Polaronic behavior in a weak-coupling superconductor. Proc. Natl. Acad. Sci. USA.

[11] Tomioka Y., Shirakawa N., Shibuya K., Inoue I.H. (2019). Enhanced superconductivity close to a non-magnetic quantum critical point in electron-doped strontium titanate. Nat. Commun..

[12] Gastiasoro M.N., Trevisan T.V., Fernandes R.M. (2020). Anisotropic superconductivity mediated by ferroelectric fluctuations in cubic systems with spin-orbit coupling. Phys. Rev. B.

[13] Yoon H., Swartz A.G., Harvey S.P., Inoue H., Hikita Y., Yu Y., Chung S.B., Raghu S., Hwang H.Y. (2021). Low-density superconductivity in SrTiO_3_ bounded by the adiabatic criterion. arXiv.

[14] Rischau C.W., Pulmannová D., Scheerer G.W., Stucky A., Giannini E., van der Marel D. (2022). Isotope tuning of the superconducting dome of strontium titanate. Phys. Rev. Res..

[15] Yu Y., Hwang H.Y., Raghu S., Chung S.B. (2022). Theory of superconductivity in doped quantum paraelectrics. NPJ Quantum Mater..

[16] Volkov P.A., Chandra P., Coleman P. (2022). Superconductivity from energy fluctuations in dilute quantum critical polar metals. Nat. Commun..

[17] Tomioka Y., Shirakawa N., Inoue I.H. (2022). Superconductivity enhancement in polar metal regions of Sr_0.95_Ba_0.05_TiO_3_ and Sr_0.985_Ca_0.015_TiO_3_ revealed by systematic Nb doping. npj Quantum Mater..

[18] Klein A., Kozii V., Ruhman J., Fernandes R.M. (2023). Theory of criticality for quantum ferroelectric metals. Phys. Rev. B.

[19] Schooley J.F., Hosler W.R., Cohen M.L. (1964). Superconductivity in Semiconducting SrTiO_3_. Phys. Rev. Lett..

[20] Koonce C.S., Cohen M.L., Schooley J.F., Hosler W.R., Pfeiffer E.R. (1967). Superconducting Transition Temperatures of Semiconducting SrTiO_3_. Phys. Rev..

[21] Prakash O., Kumar A., Thamizhavel A., Ramakrishnan S. (2017). Evidence for bulk superconductivity in pure bismuth single crystals at ambient pressure. Science.

[22] Thiemann M., Beutel M.H., Dressel M., Lee-Hone N.R., Broun D.M., Fillis-Tsirakis E., Boschker H., Mannhart J., Scheffler M. (2018). Single-Gap Superconductivity and Dome of Superfluid Density in Nb-Doped SrTiO_3_. Phys. Rev. Lett..

[23] Gastiasoro M.N., Temperini M.E., Barone P., Lorenzana J. (2022). Theory of superconductivity mediated by Rashba coupling in incipient ferroelectrics. Phys. Rev. B.

[24] van Mechelen J.L.M., van der Marel D., Grimaldi C., Kuzmenko A.B., Armitage N.P., Reyren N., Hagemann H., Mazin I.I. (2008). Electron-Phonon Interaction and charge carrier mass enhancement in SrTiO_3_. Phys. Rev. Lett..

[25] Gastiasoro M.N., Temperini M.E., Barone P., Lorenzana J. (2023). Generalized Rashba electron-phonon coupling and superconductivity in strontium titanate. Phys. Rev. Res..

[26] Fauqué B., Jiang S., Fennell T., Roessli B., Ivanov A., Roux-Byl C., Baptiste B., Bourges P., Behnia K., Tomioka Y. (2024). The polarisation fluctuation length scale shaping the superconducting dome of SrTiO_3_. arxiv.

[27] Esswein T., Spaldin N.A. (2023). First-principles calculation of electron-phonon coupling in doped KTaO_3_. Open Res. Europe.

[28] Giannozzi P., Baroni S., Bonini N., Calandra M., Car R., Cavazzoni C., Ceresoli D., Chiarotti G.L., Cococcioni M., Dabo I. (2009). QUANTUM ESPRESSO: A modular and open-source software project for quantum simulations of materials. J. Phys. Condens. Matter.

[29] Giannozzi P., Andreussi O., Brumme T., Bunau O., Buongiorno Nardelli M., Calandra M., Car R., Cavazzoni C., Ceresoli D., Cococcioni M. (2017). Advanced capabilities for materials modelling with Quantum ESPRESSO. J. Phys. Condens. Matter.

[30] Giannozzi P., Baseggio O., Bonfà P., Brunato D., Car R., Carnimeo I., Cavazzoni C., de Gironcoli S., Delugas P., Ferrari Ruffino F. (2020). Quantum ESPRESSO toward the exascale. J. Chem. Phys..

[31] Perdew J.P., Zunger A. (1981). Self-interaction correction to density-functional approximations for many-electron systems. Phys. Rev. B.

[32] Blöchl P.E. (1994). Projector augmented-wave method. Phys. Rev. B.

[33] Dal Corso A. (2014). Pseudopotentials periodic table: From H to Pu. Comput. Mater. Sci..

[34] Monkhorst H.J., Pack J.D. (1976). Special points for Brillouin-zone integrations. Phys. Rev. B.

[35] Gastiasoro M.N., Ruhman J., Fernandes R.M. (2020). Superconductivity in dilute SrTiO_3_: A review. Ann. Phys..

[36] Cancellieri C., Mishchenko A.S., Aschauer U., Filippetti A., Faber C., Barišić O.S., Rogalev V.A., Schmitt T., Nagaosa N., Strocov V.N. (2016). Polaronic metal state at the LaAlO_3_/SrTiO_3_ interface. Nat. Commun..

[37] Giustino F., Cohen M.L., Louie S.G. (2007). Electron-phonon interaction using Wannier functions. Phys. Rev. B.

[38] Poncé S., Margine E.R., Verdi C., Giustino F. (2016). EPW: Electron–phonon coupling, transport and superconducting properties using maximally localized Wannier functions. Comput. Phys. Commun..

[39] Pizzi G., Vitale V., Arita R., Blügel S., Freimuth F., Géranton G., Gibertini M., Gresch D., Johnson C., Koretsune T. (2020). Wannier90 as a community code: New features and applications. J. Condens. Matter Phys..

[40] Allen P.B. (1972). Neutron Spectroscopy of Superconductors. Phys. Rev. B.

[41] Lin X., Bridoux G., Gourgout A., Seyfarth G., Krämer S., Nardone M., Fauqué B., Behnia K. (2014). Critical Doping for the Onset of a Two-Band Superconducting Ground State in SrTiO_3-δ_. Phys. Rev. Lett..

[42] Collignon C., Fauqué B., Cavanna A., Gennser U., Mailly D., Behnia K. (2017). Superfluid density and carrier concentration across a superconducting dome: The case of strontium titanate. Phys. Rev. B.

[43] Ngai K.L. (1974). Two-Phonon Deformation Potential and Superconductivity in Degenerate Semiconductors. Phys. Rev. Lett..

[44] Kiselov D.E., Feigel’man M.V. (2021). Theory of superconductivity due to Ngai’s mechanism in lightly doped SrTiO_3_. Phys. Rev. B.

