# Supplementary material for: Effects of Homogeneous Doping on Electron–Phonon Coupling in SrTiO3
Source: Nanomaterials (Basel). 2025 Jan 17;15(2):137. doi: 10.3390/nano15020137 (PMC11767448; doi:10.3390/nano15020137)
Supplement: Supplementary file 1 [file nanomaterials-15-00137-s001.zip › nanomaterials-3396509-supplementary.pdf]

Supplementary Information for "Effects of Homogeneous Doping on Electron--Phonon Coupling in $SrTiO_3$"

Minwoo Park[1], Suk Bum Chung[1,2,†]

[1]Department of Physics and Natural Science Research Institute, University of Seoul, Seoul 02504, Republic of Korea

[2]School of Physics, Korea Institute for Advanced Study, Seoul 02455, Republic of Korea

†sbchung0@uos.ac.kr

1 STO electronic band structure fitting

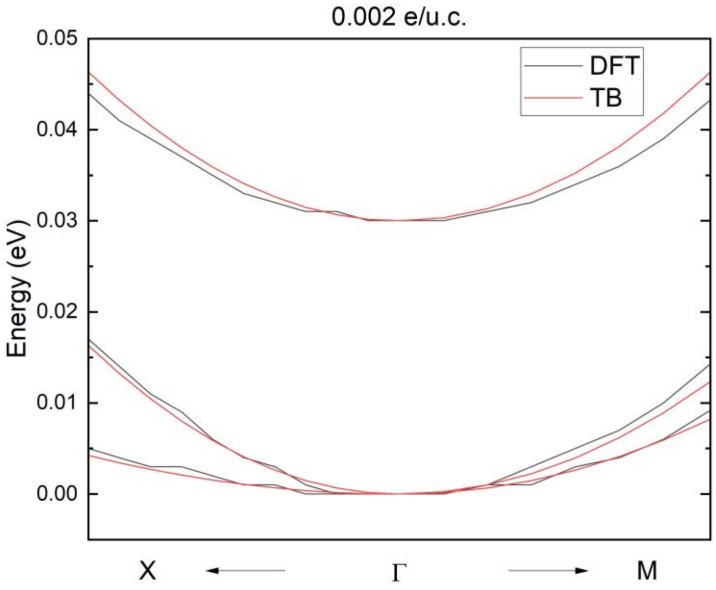

Supplementary Figure S1. Calculated band structure and tight-binding fitting near Γ point with 0.002e/u.c.

In a tight-binding model for the STO lowest conduction bands that includes hopping terms up to the next-nearest neighbor, the eigenvalues of the matrix

$$\mathcal{H}_0(\mathbf{k}) = \begin{bmatrix} \epsilon_{xx}(\mathbf{k}) & \epsilon_{xy}(\mathbf{k}) & \epsilon_{xz}(\mathbf{k}) \\ \epsilon_{yx}(\mathbf{k}) & \epsilon_{yy}(\mathbf{k}) & \epsilon_{yz}(\mathbf{k}) \\ \epsilon_{zx}(\mathbf{k}) & \epsilon_{yy}(\mathbf{k}) & \epsilon_{zz}(\mathbf{k}) \end{bmatrix} + \frac{\xi}{2} \begin{bmatrix} 0 & -i & 1 \\ i & 0 & i \\ 1 & -i & 0 \end{bmatrix}, \tag{S1}$$

where

$$\begin{aligned}\epsilon_{ij} &= \delta_{ij}\left[2t_1\sum_{\mu\neq i}(1-\cos k_\mu)+2t_2(1-\cos k_i)-4t_3\frac{\prod_\mu \cos k_\mu}{\cos k_i}\right] \\ &\quad -4t_4(1-\delta_{ij})\sin k_i \sin k_j \end{aligned} \tag{S2}$$

are the band energy eigenvalues of the Ti t_{2g} basis ($|d_{yz}\sigma\rangle, |d_{zx}\sigma\rangle, |d_{xy}\bar{\sigma}\rangle$), where $\sigma =\uparrow,\downarrow$, and $\bar{\sigma}$ signifies the spin state orthogonal to σ [1, 2].

We found that the DFT electronic band dispersion is nearly independent of the doping concentration and, from the 0.002e/u.c. dispersion plotted in Fig. S1 above, can be fitted to the tight-binding parameters $t_i(i = 1-4) = 515,172,74,42$ meV and $\xi = 20$ meV.

2 The doping evolution of the polar soft mode velocity and correlation length

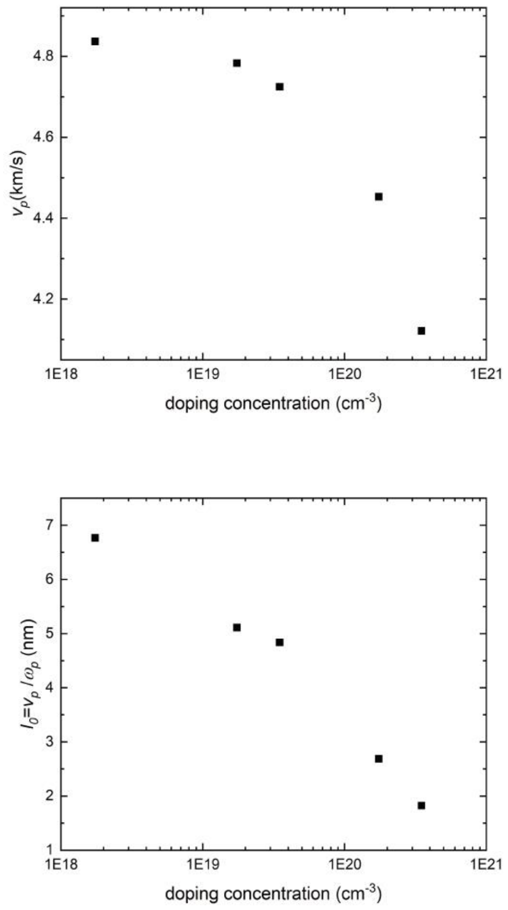

Supplementary Figure S2. The doping evolution of the velocity v_p (upper) and the correlation length $\ell_0 \equiv v_p/\omega$ (lower), respectively, of the polar soft mode. The former is obtained from fitting to $E^2(q) = \omega^2 + v_p^2 q^2$ along the [110] direction, and the latter from ω taken from Fig. 1 of the main text.

3 The doping evolution of the lattice constant

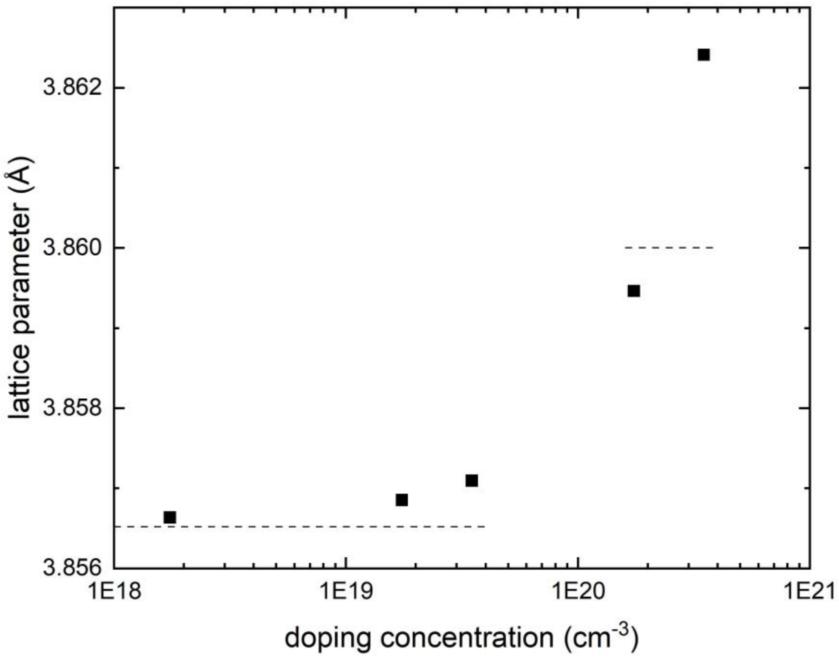

Supplementary Figure S3. The doping evolution of the lattice parameter. As explained in Sec. II of the main text, we show the lattice constant for which the Hellmann–Feynman force, *i.e.*, stress, of the cubic lattice is minimized. For comparison, the values of a_0=3.8565 ˚A and a_0=3.8600 ˚A are indicated by the two sets of horizontal dashed segments.

References

[1] M. N. Gastiasoro, J. Ruhman, and R. M. Fernandes, Superconductivity in dilute SrTiO3: A review, Ann. Phys. **417**, 168107 (2020).

[2] M. N. Gastiasoro, M. E. Temperini, P. Barone, and J. Lorenzana, Generalized rashba electron-phonon coupling and superconductivity in strontium titanate, Phys. Rev. Res. **5**, 023177 (2023).
